# Sustaining and Training for Resilience, Engagement and Meaning (STREAM): Program Model, Rationale and Early Implementation

**DOI:** 10.1186/s12909-025-07063-4

**Published:** 2025-05-17

**Authors:** Julie A. Young, Janet R. Serwint, Rachel E. M. Cramton, John D. Mahan

**Affiliations:** 1 Nationwide Children’s Hospital, Columbus, OH USA; 2 Johns Hopkins University School of Medicine, Baltimore, MD USA; 3 Arizona College of Medicine, Tucson, AZ USA

**Keywords:** Physician, Trainee, Well-being, PERMA-H, Resilience, Engagement, Joy, Meaning, Mental health

## Abstract

**Background:**

It is clear now that burnout and stress in physicians is a major threat to both physician and patient outcomes. While there are systems issues that need to be addressed, there are individual strategies that can be employed to alleviate at least some of the burden on physicians.

**Methods:**

Here we describe the development of the Sustaining and Training for Resilience, Engagement and Meaning (STREAM) program designed to support physicians in developing key skills. We utilized the PERMA-H framework to organize the program into four areas: Mental Health, Resilience, Engagement in Systems Improvement, and Connection with Joy and Meaning. We describe the rationale and need for work in these four domains to support physician well-being. Results: We detail the initial program implementation as well as the improvements we completed after gathering feedback from multiple stakeholders, including trainees, faculty, program leaders and an advisory board.

**Conclusions:**

Supporting physician well-being is paramount; building strong relationships and working together to improve systems drivers of distress are germane avenues to accomplish this goal.

## Background

Those who practice medicine typically aspire to the positive aspects of humanism in medicine striving to provide excellent care, adhering to ethical standards and contributing to the greater good. Unfortunately, multiple characteristics of our dominant medical culture, including tendency to strive for perfectionism, an exaggerated sense of responsibility, frequent self-criticism and suffering in silence, can be detrimental for physicians and lead to toxic effects such as unremitting stress and burnout [1]. The prevalence of extreme stress, burnout and more serious mental health concerns in physicians has only been exacerbated by the COVID-19 pandemic [2]. Practitioners in rural and medically underserved areas are particularly affected due to relative paucity of providers in these regions, higher rates of poverty and chronic disease in their patient populations and frequently higher case rates of COVID during the recent pandemic [3]. The impact in terms of providers delivering less effective care (including increased medical error rates) [4], experiencing personal mental health challenges, and greater rates of leaving the field of medicine is real and concerning for the providers as well as their patients and society at large. Fewer physicians are choosing to specialize in pediatrics as evidenced by recent reductions in filling residency positions [5]. The number of family medicine physicians caring for children is also declining [6] and many current pediatricians are reducing clinical hours [7]. Pediatric physicians’ rates of burnout are growing faster than other physician groups [8], highlighting the need for pediatric hospital systems to create environments where their physicians can thrive and reverse the current trends producing a shortfall of qualified physicians to care for the nation’s children and adolescents.

Ultimately, healthier physicians deliver better patient care, and are more like to continue to practice and deliver care [9]. As programs designed to improve resiliency in health care professions have been developed and enacted, dissemination of such training programs to broad groups of providers has been a challenge; moreover many resiliency training programs for physicians have been poorly received since they consist of static, online educational resources. In addition, such programs often focus on the individual provider and ignore the major negative impact on the providers of a flawed medical system and organizational challenges, which accounts for up to 80% of physician dissatisfaction and distress [10]. 

A gap exists in the availability of training programs and curricula for physicians that is grounded in evidence. As described in the literature existing trainings tend to not be comprehensive, usually focusing on a single topic. Most trainings are either stand-alone workshops or online, self-study without a virtual (live) feature for interaction. Conceptual knowledge presentation can be linked to skills-building training to more effectively develop skills in participants. Few existing trainings have been updated to reflect the racial and ethnic diversity of the physician community or health equity needs such as providers serving rural and medically underserved areas.

## Methods

In order to address both the system and individual factors which contribute to physician distress, the STREAM (*Sustaining and Training for Resilience*,* Engagement and Meaning)* training program was developed to help physicians and other health care professionals build personal skills to improve their well-being and engage in fostering system improvements. *The three residency and faculty development leadership at the institutions involved in year one were highly engaged and supportive of implementing STREAM sessions.* Participants were asked to register for the program and informed consent was included in this process. If they did not complete the registration process, they were still able to attend the program sessions.

The STREAM program was designed to include several key features:

1. A focus on skills building, in addition to acquisition of key concepts related to physician well-being.
2. The development of enduring educational methods and resources for individuals to continue their personal efforts to further develop and sustain their well-being after the training sessions.
3. Participation in a group setting to enhance social culture to reinforce learning and learn from the unique perspectives of others.

### STREAM curriculum development

In the development stage, STREAM brought together key partners, stakeholders, and subject matter experts to plan, develop, deliver, evaluate, disseminate, and sustain a comprehensive brief raining program for pediatric resident physicians and pediatric faculty incorporating evidence-based and evidence-informed approaches. We utilized the framework of Russ-Eft, who described essential elements in successful workplace training programs that included supervisor and peer support, realistic training, advanced organization, mastery orientation, spaced learning, opportunities to apply in real life, goal setting and post-training follow-up [11]. 

**Fig. 1:**
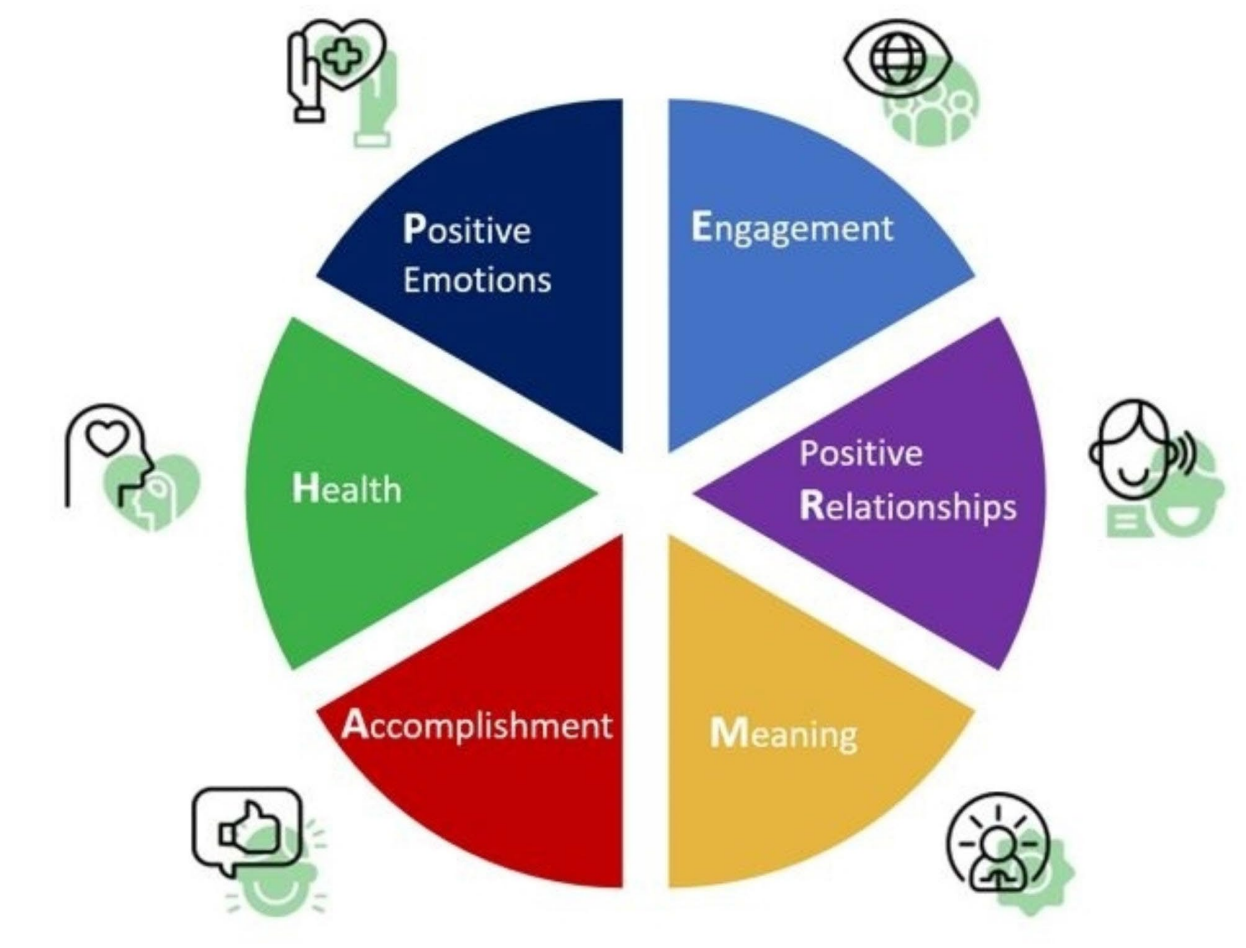
PERMA-H model well being

A variety of topics were investigated and discussed among STREAM leadership for inclusion into the program, including those identified by Shanafelt and by Swensen [12, 13]. After review, we organized the topics with high evidence into four domains: (1) Optimizing mental health, (2) building resiliency, (3) collaborative engagement to improve work and (4) connecting with joy and meaning in medicine. Because of the importance of positive collegial relationships, each domain contains information about creating and maintaining these relationships. Connections to principles such as diversity, exclusion and inclusion were also incorporated into each workshop. The conceptual well-being framework chosen to frame these training sessions, developed by Seligman and colleagues in the PERMA-H model, posits the presence of 6 key characteristics (positivity, engagement, relationships, meaning, accomplishment and health) as defining the ability of the individual to flourish and be well (Fig. 1) [14]. 

The STREAM program connects each session to specific PERMA-H activities and skills (Table 1).

**Table 1:** STREAM sections, concepts and skills

STREAM Section	PERMA-H Concepts	Skills
Mental Health	Positive Emotions Positive Relationships Health	Prioritizing mental health Supporting struggling colleagues
Resilience	Positive Emotions Meaning Positive Relationships	Positive Psychology Practice
Engagement	Engagement Accomplishment Positive Relationships	Work Improvement
Joy and Meaning	Positive Emotions Positive Relationships Meaning	Reflective Practice

### Optimizing mental health

Concerns of poor mental health among healthcare professionals are rising. In most studies, 40–50% of physicians endorse burnout symptoms [8]. For trainees, rates of burnout peak during residency and fellowship training, and in pediatric residents, burnout prevalence is over 50% [15]. The COVID-19 pandemic brought to the forefront the widespread burnout prevalence and its consequences. Beyond burnout, depression is also common in physicians, with 28% of residents meeting criteria for major depression during their training [16]. Physicians have the highest suicide rate of any profession, reportedly 2–3 times that of the general. Programs that address well-being and burnout may reduce suicide rates in physicians [17]. Unfortunately, many physicians with mental health problems do not seek help and do not realize they are at risk [18], resulting in these conditions being underdiagnosed and untreated. Physicians commonly neglect their own health and experience barriers to care [19]. Appropriately addressing those with serious mental health concerns involves both identifying those at risk and providing timely assistance. Resident training is a particularly important time to attend to mental health issues due to issues of medical culture and perfectionism in addition to the stresses of striving to gain competence, with long hours and a unique position within the health care system [15]. 

The key skill addressed by this domain is the concept of treating *mental health as a vital sign;* underscoring the belief that attention to mental health should be a deliberate priority for individual physicians, teams, healthcare institutions and systems. *Mental health exists on a continuum from severe diagnosable conditions to thriving. While there are specific interventions (i.e. Cognitive Behavioral Therapy) that are well established as effective treatments for mental health conditions*,* there are many strategies that can be utilized to support positive mental health for the majority of the population.* This session teaches participants strategies that can support positive mental health such as gratitude and self-compassion as well as providing an opportunity to practice a supportive conversation with a potentially struggling colleague. The connections to the PERMA-H model in this domain are positive emotions, positive relationships and health. Strategies such as gratitude and relational pauses are discussed as both individual interventions, such as creating a well-being plan and as structural ones, such as debreifings.

### Building resilience

In addition to the critical importance of prioritizing mental health, STREAM focuses on providing physicians with strategies to support flourishing. Resilience is a process which allows a person to adapt to challenging circumstances and remain hopeful [20]. There are a variety of ways to build resilience and even small gains in resilience are meaningful [21]. This is promising, given that many physicians have moderate to high resilience [22, 23]. Professional resilience is promoted by training in well-being principles, positive psychology, pragmatic mindfulness skills, positivity, and value congruence in the workplace. Promoting gratitude techniques and self-compassion have been linked to well-being. In a recent Pediatric Resident Burnout-Resilience Study Consortium (PRB-RSC) study, self-compassion was protective for burnout, stress and ability to deliver calm, compassionate care [24]. Emotional debriefings provide opportunities for teams to participate in group reflection following a sentinel event or challenging experience [25]. Stimulating pediatricians to engage in organizational efforts to improve the workplace and promote a culture of well-being is associated with better job satisfaction and job retention [26]. 

A key focus in the STREAM Resilience domain is positive psychology. Positive psychology interventions have been shown to improve resilience in physicians [23, 27]. This STREAM workshop allows participants to assess their current state relative to positive and negative emotions and practical suggestions on keeping their perspectives positive. The connections to the PERMA-H model include positive emotions, positive relationships, and meaning. Reflecting on values and promoting positivity can impact physician resilience.

### Collaborative engagement to improve work

Physician engagement is a term that is widely used and has a number of definitions in the literature [28]. In STREAM, engagement is posited as being actively involved with workplace improvement. Masloch’s full theory offers insights into moving towards engagement by modifying rewards, control, workload, fairness/transparency, community and values [29]. Engagement in work is associated with several benefits for the physician, including greater opportunities for meaning and joy, but especially improving the micro-environment (and the health care team’s work experience), providing greater work satisfaction, and deeper relationships with colleagues involved in improvement efforts. Engagement has been posited as the opposite of burnout; those who are not burned out are more likely to be satisfied with their job and less likely to consider leaving the profession or experience career regret [4]. Physicians more engaged in their work have lower levels of burnout and are more likely to endorse plans to stay in that position and institution during the next year. Engaged physicians have positive perceptions of workload, control and community (including positive feedback and supervisor support) [30, 31]. These three concepts are covered in the Engagement workshops.

This domain’s workshops cover this somewhat ‘counter-intuitive’ concept and suggest a variety of work improvement options, including efforts to improve clinic and in patient processes, develop care guidelines, new services, and quality improvement efforts. While many participants may be engaged in such activities at the time of the workshop, the emphasis is on increasing engagement, especially with colleagues, and insuring support from leadership for improvement efforts.

The skill in in this domain focuses on work improvement. The connections to the PERMA-H model include accomplishment, engagement, and positive relationships.

### Connecting with joy and meaning in medicine

Helping pediatricians and residents recapture or maintain their joy and meaning in medicine can help support physician well-being, professional satisfaction, and a healthy work environment [32, 33]. Joy can be conceptualized as pleasure and satisfaction about how and why one is a physician. In this sense, joy is an internal barometer of well-being and wellness. Joy is substantially different than happiness. Happiness is typically derived from gaining things and accomplishments. Joy is that pleasure of being, becoming and doing.

As Grimes explains, the physician who is able to seek and find satisfaction in the work, spend time on meaningful pursuits, and retain connection to what is important is more likely to remain joyful, and be resilient in the face of setbacks [34]. Discussion and appreciation of joy in the work, patient benefits, gratitude, and satisfaction in work well done can promote joy in the profession and the work. Well-being is promoted in those who are able harness joy available in this work.

The connection to the PERMA-H model in this domain include positive emotions, positive relationships, and meaning.

## Results

### STREAM program development

Key stakeholder groups were formed to develop interactive content that would promote multiple learning opportunities in each domain. The STREAM Leadership Team included subject matter experts for each of the four key areas, a medical education development specialist and an expert in incorporating DEI concepts in medical education. In addition, the PRB-RSC leadership team served as the STREAM Faculty Advisory Group to assess the validity and quality of the content for each domain. The Resident Advisory Committee was formed of interested residents at the three initial sites to ensure content resonated with medical learners. All session design groups (content experts) groups had multiple meetings with both the educational specialist and the DEI expert during the design process. All initial sessions were recorded prior to presentations and each of these groups viewed and critiqued the content before the workshop content was finalized for year 1 implementation. *STREAM leadership*,* in consultation with the Faculty Advisory Group and the Resident Advisory Committee determined that the primary program outcomes were participation*,* collaborative learning and skill practice.*

### Program implementation

Training sites were identified through PRB-RSC website, an organization of pediatric residency programs across the country who have been working to improve well-being in pediatric residents since 2012. The populations served by the institutions in the PRB-RSC involve urban underserved populations, rural groups, and children, a group who comprise half of Medicaid enrollees. With PRB-RSC members representative of the widespread national distribution of all pediatric training programs, the STREAM program was designed to address mental health issues and build the resiliency and strengths of physicians who care for the broad range of children in the nation.

The STREAM leadership team created a website with a learning management system (LMS) to track participation and to provide additional educational materials for those enrolled. STREAM leadership committed to posting a monthly blog around a topic relevant to STREAM and creating supplementary learning resources to support growth in each of the four domains.

In the first year (2022), STREAM’s comprehensive brief training program utilized a sequence of 8 synchronous (live) virtual one-hour sessions led by trained facilitators from the STREAM leadership team and also provided access to a menu of complementary short online modules to extend learning and skill development in these areas with training and CEUs provided at no cost to trainees. The three core clinical training sites (Nationwide Children’s Hospital, Colorado Children’s Hospital, and Seattle Children’s Hospital) implemented the program that year. The curriculum addressed training interventions and efficiencies in organizational culture in key domains and is organized into four key training pillars: *Attending to Your Mental Health; Professional Resilience; Collaborative Engagement to Improve Your Work; Connecting with Joy and Meaning in Medicine.* A significant protocol and system-wide approach was deployment of the evidence-based intervention, the Interactive Screening Program (ISP) at no cost to sites. STREAM was formulated to empower pediatricians to address system issues through training in practical methods to support improvement efforts, such as participating in quality improvement projects for better outcomes for patients and institution, working on Electronic Health Records optimization and initiatives to promote positive team culture. The train-the-trainer curriculum and model and the training pillars (curriculum) were included in the products which are being evaluated and contribute to sustainability.

Key milestone activities were phased and scaled in order to assure quality of the training program and to build sustainability while assuring the rapid response required by current conditions. In the first year, we developed and delivered the curriculum using the three committed sites. These sites each had a domain leader. Additional sites were added in cohorts in the second and third years with original sites continuing on in the program. Across the three sites, we enrolled 117 faculty and 37 trainees. An early challenge noted was participation attendance at noon sessions, particularly with residents. Those that attended provided positive feedback for the importance and relevance of the sessions. After reviewing all participant feedback from the three initial sites, several key principles were adopted as we modified the curriculum for year two.

1. STREAM sessions were condensed to 60 and 90-minute versions and each could stand alone or be presented as a series of the four topics mentioned above.
2. STREAM sessions were offered during normally scheduled meetings, retreats, academic days, etc. Sessions would be preferentially scheduled during in-person meetings, though virtual presentations could continue.
3. Each STREAM session was focused on skill-building. Time to practice the skills would be embedded into the session and each session would have 30–50% interactive components.
4. STREAM sessions explicitly called out the systems issues in healthcare and offer practical ways to use the skills from the sessions at both the individual and systems level.
5. Each STREAM session explicitly covered an element of DEI and connected it to the topic.

Additionally in year one, we created a training institute with the help of our medical educational specialist. Training facilitators at new sites consisted of three sessions covering STREAM background and purpose, effective facilitation, STREAM content and interactive components. We recorded our presenters from year one in short (less than five minute) sections and embedded them into PowerPoint presentation decks. This allowed for STREAM faculty at each institution to focus on leading the interactive portions of the program without having to become subject matter experts in all four areas.

## Conclusions

Promoting physician well-being is essential for our health care system, both the physicians as individuals and the patients for whom they care. Individual skill-building should be promoted due the immediacy of impact while expanding attention to improve work systems and environments. Individuals who make relatively small gains in induvial well-being may be able to more effectively work to change the systems drivers of distress that plague our current medical system. Additionally, even incremental gains in individual well-being can be clinically relevant. Because systems-issues are large drivers of distress and burnout for physicians, interventions targeting healthcare systems are imperative for the sustainability of the physician workforce.

## Data Availability

The data used during the current study are available from the corresponding author on reasonable request. These data include number of participants consenting to participate in year one as well as responses to questions related to improving the content and implementation of the program.

## References

[1] Stewart MT, Serwint JR. Burning without burning out: A call to protect the calling of medicine. Curr Probl Pediatr Adolesc Health Care. 2019;49(11):100655. 31631025 10.1016/j.cppeds.2019.100655

[2] Bhardwaj A. COVID-19 pandemic and physician burnout: ramifications for healthcare workforce in the united States. J Healthc Leadersh. 2022;14:91–7. 35726282 10.2147/JHL.S360163 PMC9206033

[3] Anaraki NR, Mukhopadhyay M, Karaivanov Y, Wilson M, Asghari S. Living and working in rural healthcare during the COVID-19 pandemic: a qualitative study of rural family physicians’ lived experiences. BMC Prim Care. 2022;23(1):335. 36550406 10.1186/s12875-022-01942-1 PMC9773678

[4] Hodkinson A, Zhou A, Johnson J, Geraghty K, Riley R, Zhou A, Panagopoulou E, Chew-Graham CA, Peters D, Esmail A, Panagioti M. Associations of physician burnout with career engagement and quality of patient care: systematic review and meta-analysis. BMJ. 2022;378:e070442. 36104064 10.1136/bmj-2022-070442 PMC9472104

[5] Results and Data.: 2024 Main Residency Match [https://www.nrmp.org/match-data/2024/06/results-and-data-2024-main-residency-match/]

[6] Vinci RJ. The pediatric workforce: recent data trends, questions, and challenges for the future. Pediatrics 2021, 147(6). 10.1542/peds.2020-013292 33692163

[7] Rivara FP, Gonzalez-Del-Rey J, Forrest CB. The pediatric subspecialty physician workforce. JAMA Pediatr. 2024;178(2):107–8. 38109094 10.1001/jamapediatrics.2023.5235

[8] Shanafelt TD, Hasan O, Dyrbye LN, Sinsky C, Satele D, Sloan J, West CP. Changes in burnout and satisfaction with Work-Life balance in physicians and the general US working population between 2011 and 2014. Mayo Clin Proc. 2015;90(12):1600–13. 26653297 10.1016/j.mayocp.2015.08.023

[9] Shanafelt TD, Noseworthy JH. Executive leadership and physician Well-being: nine organizational strategies to promote engagement and reduce burnout. Mayo Clin Proc. 2017;92(1):129–46. 27871627 10.1016/j.mayocp.2016.10.004

[10] Collier R. Addressing physician burnout at the systems level. CMAJ. 2018;190(6):E174. 29440342 10.1503/cmaj.109-5556 PMC5809223

[11] Russ-Eft DF, Preskill HS. Evaluation in Organizations: A Systematic Approach to Enhancing Learning, Performance, and Change. In: *2001*; 2001.

[12] Swensen SJ, Shanafelt T. An organizational framework to reduce professional burnout and bring back joy in practice. Jt Comm J Qual Patient Saf. 2017;43(6):308–13. 28528625 10.1016/j.jcjq.2017.01.007

[13] Shanafelt TD, Wang H, Leonard M, Hawn M, McKenna Q, Majzun R, Minor L, Trockel M. Assessment of the association of leadership behaviors of supervising physicians with Personal-Organizational values alignment among staff physicians. JAMA Netw Open. 2021;4(2):e2035622. 33560424 10.1001/jamanetworkopen.2020.35622 PMC7873777

[14] Seligman M. Flourish: A visionary new Understanding of happiness and well-being. New York, NY: Free; 2011.

[15] Kemper KJ, Schwartz A, Wilson PM, Mahan JD, Schubert CJ, Staples BB, McClafferty H, Serwint JR, Batra M. Pediatric resident burnout-Resilience study C: burnout in pediatric residents: three years of National survey data. Pediatrics 2020, 145(1). 10.1542/peds.2019-1030 31843859

[16] Rotenstein LS, Ramos MA, Torre M, Segal JB, Peluso MJ, Guille C, Sen S, Mata DA. Prevalence of depression, depressive symptoms, and suicidal ideation among medical students: A systematic review and Meta-Analysis. JAMA. 2016;316(21):2214–36. 27923088 10.1001/jama.2016.17324 PMC5613659

[17] Kalmoe MC, Chapman MB, Gold JA, Giedinghagen AM. Physician suicide: A call to action. Mo Med. 2019;116(3):211–6. 31527944 PMC6690303

[18] Slat EA, Parsley IC, Gold JA. Recognizing decline in physician wellbeing: when to seek help or intervene. Mo Med. 2021;118(6):494–8. 34924610 PMC8672954

[19] George S, Hanson J, Jackson JL. Physician, heal thyself: a qualitative study of physician health behaviors. Acad Psychiatry. 2014;38(1):19–25. 24464415 10.1007/s40596-013-0014-6

[20] Physical Activity Guidelines for American, 2nd edition. 2018.

[21] Mahmoud NN, Rothenberger D. From burnout to Well-Being: A focus on resilience. Clin Colon Rectal Surg. 2019;32(6):415–23. 31686993 10.1055/s-0039-1692710 PMC6824889

[22] Velasquez Marin CA, Avendano-Vasquez CJ. Anxiety and resilience in palliative medicine physicians. BMJ Support Palliat Care 2024. 10.1136/spcare-2023-004455 38955460

[23] West CP, Dyrbye LN, Sinsky C, Trockel M, Tutty M, Nedelec L, Carlasare LE, Shanafelt TD. Resilience and burnout among physicians and the general US working population. JAMA Netw Open. 2020;3(7):e209385. 32614425 10.1001/jamanetworkopen.2020.9385 PMC7333021

[24] Liu A, Ben-Zion S, Schwartz A, Mahan JD, Reed S. Well-being factors associated with confidence in providing calm, compassionate care in pediatric residents. Patient Educ Couns. 2023;115:107906. 37478547 10.1016/j.pec.2023.107906

[25] Osta AD, King MA, Serwint JR, Bostwick SB. Implementing emotional debriefing in pediatric clinical education. Acad Pediatr. 2019;19(3):278–82. 30343057 10.1016/j.acap.2018.10.003

[26] Wilson PM, Batra M, Kemper KJ, Mahan JD, Staples BB, Serwint JR. Physician Well-being. Pediatr Rev. 2019;40(Suppl 1):12–20. 31575686 10.1542/pir.2018-0329

[27] Townsley AP, Li-Wang J, Katta R. Healthcare workers’ Well-Being: A systematic review of positive psychology interventions. Cureus. 2023;15(1):e34102. 36843822 10.7759/cureus.34102 PMC9946896

[28] Duggan EW, Clark M. Moving past burnout, looking toward engagement. Anesthesiol Clin. 2022;40(2):399–413. 35659410 10.1016/j.anclin.2022.01.012

[29] Baugh JJ, Takayesu JK, White BA, Raja AS. Beyond the Maslach burnout inventory: addressing emergency medicine burnout with Maslach’s full theory. J Am Coll Emerg Physicians Open. 2020;1(5):1044–9. 33145555 10.1002/emp2.12101 PMC7593437

[30] Uong AM, Cabana MD, Serwint JR, Bernstein CA, Schulte EE. Pediatric faculty engagement and associated areas of worklife after a COVID19 surge. J Healthc Leadersh. 2023;15:375–83. 38046535 10.2147/JHL.S410797 PMC10693203

[31] Solms L, van Vianen AEM, Koen J, Kan KJ, de Hoog M, de Pagter APJ, Improve Research N. Physician exhaustion and work engagement during the COVID-19 pandemic: A longitudinal survey into the role of resources and support interventions. PLoS ONE. 2023;18(2):e0277489. 36724165 10.1371/journal.pone.0277489 PMC9891506

[32] West CP, Dyrbye LN, Shanafelt TD. Physician burnout: contributors, consequences and solutions. J Intern Med. 2018;283(6):516–29. 29505159 10.1111/joim.12752

[33] Wohleverl A. Recapturing joy in medicine. Maitland, FL: Xulon; 2019.

[34] Grimes PE. Physician burnout or joy: rediscovering the rewards of a life in medicine(). Int J Womens Dermatol. 2020;6(1):34–6. 32042882 10.1016/j.ijwd.2019.12.001 PMC6997840

